# PTH-rP and PTH-R1 Expression in Placentas from Pregnancies Complicated by Gestational Diabetes: New Insights into the Pathophysiology of Hyperglycemia in Pregnancy

**DOI:** 10.3390/diagnostics11081356

**Published:** 2021-07-28

**Authors:** Angelo Sirico, Marco Dell’Aquila, Linda Tartaglione, Sascia Moresi, Giorgia Farì, Dario Pitocco, Vincenzo Arena, Antonio Lanzone

**Affiliations:** 1 Obstetrics and High-Risk Pregnancy Unit, Department of Woman, Child Health and Public Health, Fondazione Policlinico Universitario A. Gemelli IRCCS, Largo Agostino Gemelli 8, 00168 Rome, Italy; sascia.moresi@policlinicogemelli.it (S.M.); antonio.lanzone@policlinicogemelli.it (A.L.); 2 Pathology Unit, Department of Woman, Child Health and Public Health, Fondazione Policlinico Universitario A. Gemelli IRCCS, Largo Agostino Gemelli 8, 00168 Rome, Italy; mzrk07@gmail.com (M.D.); vincenzo.arena@policlinicogemelli.it (V.A.); 3 Diabetology Unit, Fondazione Policlinico Universitario A. Gemelli IRCCS, Largo Agostino Gemelli 8, 00168 Rome, Italy; linda.tartaglione@policlinicogemelli.it (L.T.); dario.pitocco@policlinicogemelli.it (D.P.); 4 Institute of Biochemistry and Clinical Biochemistry, Fondazione Policlinico Universitario A. Gemelli IRCCS, Largo Agostino Gemelli 8, 00168 Rome, Italy; angelo0857@yahoo.it; 5 Faculty of Medicine and Surgery, Università Cattolica del Sacro Cuore, Largo Agostino Gemelli 8, 00168 Rome, Italy

**Keywords:** gestational diabetes, PTH, placenta, obesity, immunochemistry, pregnancy

## Abstract

Background: this study investigated the expression of parathyroid hormone-related protein (PTH-rP) and PTH/PTH-rP receptor PTH-R1 in placentas from women with gestational DM (GDM), and the relationship between PTH-R1 and PTH-rP expression and pregnancy characteristics. Methods: we prospectively enrolled 78 pregnant women with GDM, and immunochemistry for PTH-rP and PTH-R1 was performed on placentas. Patients were grouped according to the positivity of PTH-R1 or PTH-rP expression, and pregnancy characteristics were compared between the two groups. Results: PTH-rP and PTH-R1 expression were highest in the extravillous cytotrophoblast and in the decidua. In extravillous cytotrophoblast, PTH-rP expression was higher in women with abnormal at fasting glycemia compared to women with abnormal 60′ or 120′ glycemia (25/25, 50% vs. 6/28, 21.4%, χ2 = 6.12, *p* = 0.01), and PTH-R1 expression was higher in women with abnormal oral glucose tolerance test (OGTT) at fasting glycemia compared to women with abnormal 60′ or 120′ glycemia (37/50, 74% vs. 15/28, 53.6%, χ2 = 3.37, *p* = 0.06). In syncytiotrophoblast, PTH-rP-positive placentas were characterized by higher incidence of 1 min Apgar score < 7 (2/9, 22.2% vs. 2/69, 2.9%, χ2 = 6.11, *p* = 0.01) and maternal obesity (4/9, 44.4% vs. 11/69, 16.7%, χ2 = 3.81, *p* = 0.05). Conclusion: placental PTH-rP and PTH-R1 expression is dependent on the type of maternal hyperglycemia, and it is associated with adverse pregnancy outcomes.

## 1. Introduction

Diabetes represents a clinical challenge, especially in pregnant women, where it is crucial to monitor and assess both the maternal and the fetal wellbeing. Apart from women with pregestational diabetes (type 1 or type 2 diabetes mellitus or DM), a growing rate of women develop gestational diabetes mellitus (GDM), considered as a glucose intolerance diagnosed for the first time in non-diabetic pregnant women and that typically resolves after delivery [1,2].

Specific risks of uncontrolled hyperglycemia in pregnancy include spontaneous abortion, pre-eclampsia, fetal demise, macrosomia, neonatal hypoglycemia, and hypocalcemia, among others [3,4].

Hyperglycemia in pregnancy is responsible for peculiar changes in the fetoplacental unit. Placentas from women with hyperglycemia are characterized by increased expression of glucose transporters (GLUT) GLUT1 and GLUT3 in the basal syncytiotrophoblast. In these cases, the placenta acts as a regulating glycemic buffer: once within the fetal circulation, glucose is used to cover acute fetal metabolic and energy demands [3]. The proportion of glucose that is not metabolized is then stored in various fetal tissues, predominantly the liver, heart, and skeletal muscle. When further glucose overload cannot be channeled into glycogen, the excess fetal glucose will then be taken up by the placental endothelium and metabolized, converting into glycogen. Furthermore, maternal hyperglycemia and hyperinsulinemia lead to an increased tissue oxygen consumption, which is responsible for a chronic hypoxemia in the fetoplacental unit and the subsequent upregulation of hormones (erythropoietin, fibroblast growth factor 2, Leptin, insulin-like growth 2) and inflammatory cytokines (interleukin-6, tumor necrosis factor α) that may lead to placental neoangiogenesis and hypervascularization.

During human pregnancy, parathyroid hormone-related protein (PTH-rP) and parathyroid hormone (PTH)/PTH-rP receptors are produced by the uterus, placenta, fetal membranes (amnion and chorion), and developing fetus [4]. PTH-rP has important roles in fetal growth and development through stimulation of placental calcium transport, vasodilatation of the uteroplacental vasculature, and regulation of cellular growth and differentiation [5].

Studies in normal pregnancies have demonstrated that median PTH serum concentrations are statistically lower in early pregnancy compared to late pregnancy, while PTH-rP and corrected calcium levels are significantly higher in pregnant women than in nonpregnant women [6,7]. Furthermore, it has been demonstrated that type 2 diabetics presented higher serum PTH-rP levels than control subjects, and PTH-rP induces insulin expression in pancreatic β-cells [8,9]. However, the role of PTH and PTH-rP in pregnancies complicated by GDM still remains unclear.

Thus, the aim of the present study was to investigate the expression of PTH-rP and PTH/PTH-rP receptor PTH-R1 in placentas from women with GDM and the relationship between PTH-R1 and PTH-rP expression and the obstetric outcomes.

## 2. Materials and Methods

We prospectively enrolled pregnant women with a diagnosis of GDM who delivered in our center between February and May 2018. We included in our analysis singleton nondiabetic pregnant women who were diagnosed with GDM after a 75 g oral glucose tolerance test (OGTT) at 24–28 weeks, according to the International Association of the Diabetes and Pregnancy Study Groups criteria [10]. Exclusion criteria were: presence of pregestational diabetes, chromosomal or congenital anomalies, abnormal first or second trimester ultrasound screening. Gestational age was assessed by last menstrual period, if in agreement of 7 days with crown-rump length dating using Robinson formula, or by CRL if there was more than 7 days discrepancy. For each patient, we collected the following data: maternal age, pregestational weight and body mass index (BMI), parity, presence of GDM in a previous pregnancy, insulin or diet therapy, pattern of abnormal OGTT (abnormal fasting or 1–2 h glycemia), third-trimester ultrasound-estimated fetal weight percentile for gestational week, gestational week at delivery, type of delivery, indication of caesarean section (CS) or operative vaginal delivery (OVD), neonatal sex, neonatal birthweight and birthweight percentile according to the gestational week, placental weight, fetoplacental weight ratio, presence of neonatal respiratory distress syndrome (RDS), Apgar score, neonatal admission to NICU or neonatal ward.

After delivery, placentas from included women were sent for histopathological examination. Samples were formalin-fixed and paraffin-embedded (FFPE), and, subsequently, sections were cut at 4 µm and subsequently stained with hematoxylin and eosin for routine histopathological examination. Afterwards, immunohistochemistry for PTH-rP and PTH-R1 was performed on 5 µm sections that were fixed on positively charged glass microscope slides. Sections were then deparaffinized in xylene, washed in ethanol at progressively lower concentration, and then washed with deionized water. Immunohistochemistry was performed with an anti-PTH-rP antibody and with an anti-PTH-R1 antibody. The anti-PTH-rP antibody (PTH-RP 1–34, rabbit, Yanaihara Institute, Sizuoka, Japan) was diluted at 1:100; the anti-PTH-R1 antibody (3d11 clone, mouse monoclonal, Santa Cruz biotechnology Inc., Dallas, TX, USA) was diluted at 1:100. Antigen retrieval for both the antibodies was performed with high pH with the EnVision FLEX/HRP visualization system (Dako, Agilent Technologies, Santa Clara, California, USA). Both the hematoxylin and eosin-stained sections, and those stained with the immunohistochemistry for PTH-rP and PTH-R1, were analyzed with an optical microscope by two expert pathologists in double blind.

Patients were grouped according to the positivity of PTH-R1 or PTH-rP expression in each placental layer, and maternal and ultrasound parameters were compared between the two groups. The Shapiro–Wilk test was performed to test for normality. When normally distributed, continuous variables were compared by Student’s *t*-test, otherwise by Kruskal–Wallis test; categorical variables were compared by Pearson chi-square test. A *p* value <0.10 was considered as significant. Statistical analysis was carried out using the Statistical Package for Social Sciences (SPSS) Statistics v. 19 (IBM Inc., Armonk, NY, USA).

## 3. Results

### 3.1. Expression of Parathyroid Hormone-Related Protein (PTH-rP) and PTH-R1 according to Placental Layers

**Figure 1:**
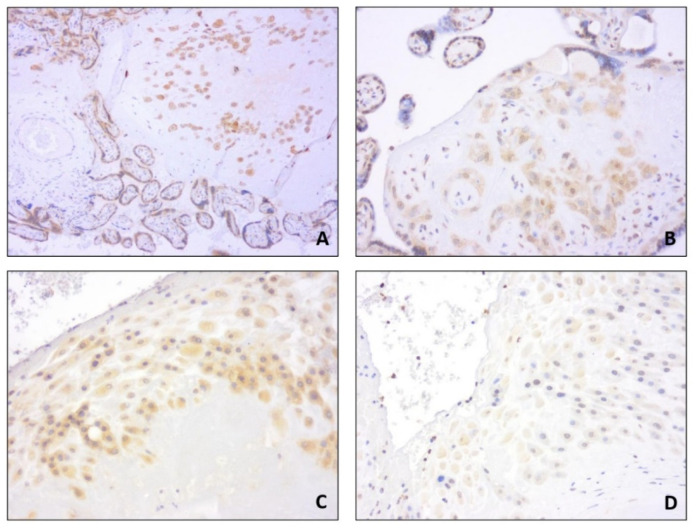
PTH-R1 (**A**) and parathyroid hormone-related protein (PTH-rP) (**B**) expression in extravillous cytotrophoblast; PTH-R1 (**C**) and PTH-rP (**D**) expression in decidua.

**Figure 2:**
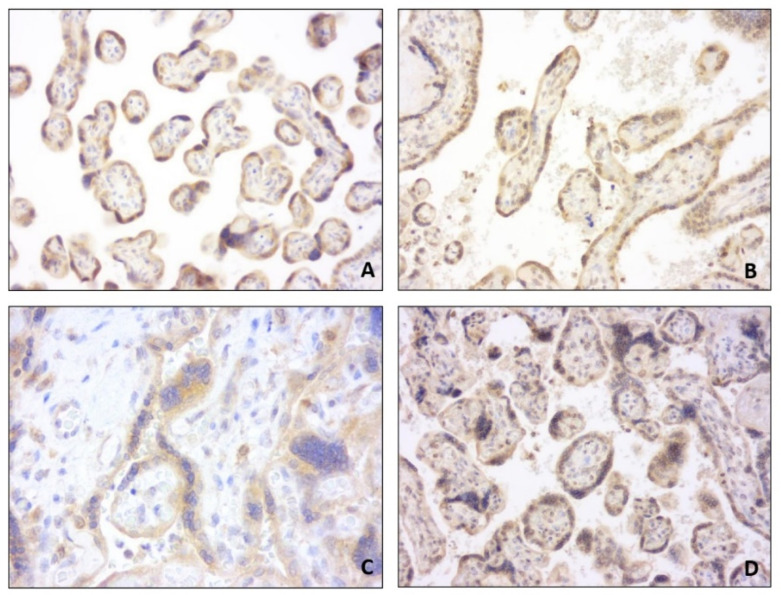
PTH-R1 (**A**) and PTH-rP (**B**) expression in villous cytotrophoblast; PTH-R1 (**C**) and PTH-rP (**D**) expression in syncytiotrophoblast.

We included in our analysis 78 patients matching the inclusion criteria. Maternal and ultrasound characteristics are summarized in Table 1. Among continuous variables, maternal age, neonatal birthweight, and placental weight showed a parametric distribution; pregestational weight and BMI, third-trimester ultrasound-estimated fetal weight percentile, gestational week at delivery, fetoplacental weight ratio, and neonatal birthweight percentile showed a nonparametric distribution. Immunochemistry analysis showed that PTH-rP expression was highest in the extravillous cytotrophoblast (31/78, 39%) and in the decidua (29/78, 37.2%) (Table 2). Similarly, PTH-R1 expression was highest in the extravillous cytotrophoblast (52/78, 66.7%) and in the decidua (58/78, 74.4%) (Figure 1). Mild expression of PTH-rP PTH-R1 was found in the villous cytotrophoblast and the syncytiotrophoblast, while no PTH-R1 positivity was found in the villous stroma (Figure 2).

### 3.2. Expression of PTH-rP and PTH-R1 according to Pregnancy Characteristics

**Figure 3:**
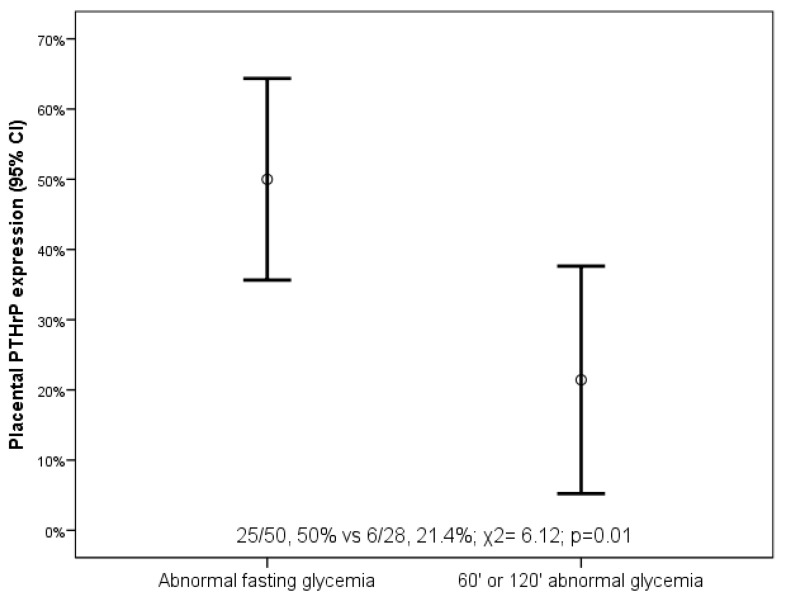
PTH-rP expression in extravillous cytotrophoblast in women with gestational diabetes according to type of abnormal glycemia at oral glucose tolerance test.

**Figure 4:**
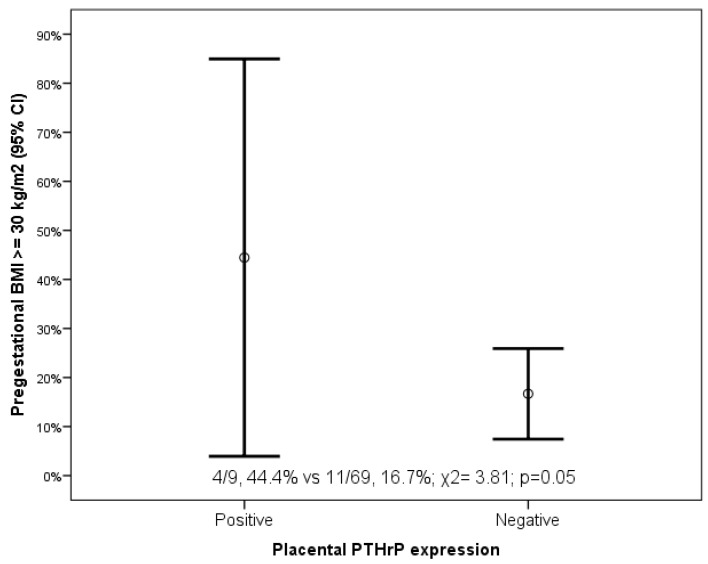
PTH-rP expression in syncytiotrophoblast in women with gestational diabetes according to maternal pregestational body mass index (BMI).

In extravillous cytotrophoblast, we found a significant difference in the rate of PTH-rP expression in women with abnormal OGTT at fasting glycemia compared to women with abnormal 60′ or 120′ glycemia (25/50, 50% vs. 6/28, 21.4%, χ2 = 6.12, *p* = 0.01) (Figure 3 and Table S1). In extravillous cytotrophoblast, we also found that PTH-R1 expression was higher in women with abnormal OGTT at fasting glycemia compared to women with abnormal 60′ or 120′ glycemia (37/50, 74% vs. 15/28, 53.6%, χ2 = 3.37, *p* = 0.07) (Figure 4). PTH-R1 expression in extravillous cytotrophoblast was associated with lower median third-trimester ultrasound-estimated fetal weight percentile (55, IQR 48.75 vs. 79.5, IQR 28, *p* = 0.02) and lower mean fetoplacental weight ratio (5.71, SD 1.53 vs. 6.34, SD 1.20, *p* = 0.04) compared to PTH-R1-negative placentas (Table S2). Interestingly, 5/52 (9.6%) pregnancies with PTH-R1-positive placenta had urgent CS for non-reassuring CTG trace, while 0/26 (0%) pregnancies with PTH-R1-negative placenta had urgent CS for non-reassuring CTG trace, even if this difference did not reach significance (risk difference 9.6, *p* = 0.16).

In syncytiotrophoblast, we found that PTH-rP-positive placentas were characterized by higher incidence of neonatal Apgar score at 1 min < 7 (2/9, 22.2% vs. 2/69, 2.9%, χ2 = 6.11, *p* = 0.01) and maternal obesity (4/9, 44.4% vs. 11/69, 16.7%, χ2 = 3.81, *p* = 0.05) (Figure 4 and Table S3). In the syncytiotrophoblast, no differences in the maternal and neonatal variables were found according to the expression of PTH-R1 (Table S4). In the villous trophoblast and in the decidua, no differences in the maternal and neonatal variables were found according to the expression of PTH-rP or PTH-R1 (Tables S5–S8).

## 4. Discussion

Our results show that, in pregnancies complicated by gestational diabetes, PTH-rP and PTH-R1 are strongly expressed in extravillous cytotrophoblast and in the decidua, mildly expressed in the syncytiotrophoblast and in the villous cytotrophoblast, while no expression of these receptors was found in the villous stroma. Placental PTH-rP and PTH-R1 expression in the extravillous cytotrophoblast is associated with increased incidence of maternal abnormal fasting glycemia at 24–28 weeks OGGT, PTH-R1 expression in the extravillous cytotrophoblast is associated with lower median third-trimester ultrasound-estimated fetal weight percentile and lower fetoplacental weight ratio, while PTH-rP expression in the syncytiotrophoblast is associated with higher incidence of maternal obesity and neonatal Apgar score at 1 min < 7.

PTH-rP was first identified in 1987 as a tumor product that had the ability to activate PTH receptors and cause hypercalcemia by increasing bone resorption and renal tubular resorption of calcium, with a phosphaturic action also, by interacting with a common PTH/PTH-rP receptor, now called type 1 PTH receptor or PTH-R1 [11,12]. In contrast to PTH, which is found only in the parathyroid glands, PTH-rP is found in many tissues in both fetuses and adults, including epithelia, mesenchymal tissues, the fetoplancental unit, endocrine glands, and the central nervous system. PTH is a circulating hormone that carries signals from a calcium sensor in the parathyroid glands to the target tissues, while PTH-rP is a local messenger within tissues [13]. Furthermore, PTH-rP is processed into at least three fragments, suggesting that it is a polyhormone, the precursor of multiple biologically active peptides. Recent findings show that PTH-rP can act, not only via the classical autocrine/paracrine pathways, but also through a so-called intracrine pathway, which involves the translocation of the nascent protein into the nucleus [14,15,16].

PTH-rP plays a crucial role in the regulation of calcium transport from the mother to the fetus through the placenta. The fetal skeleton mineralization and the calcium requirements for all the enzymatic processes in the fetal development explain the high demands of calcium during fetal growth. Therefore, a calcium gradient from the mother to the fetus is maintained through the syncytiotrophoblast via passive mechanisms, Na^+^/Ca^2+^ exchangers, and calcium binding proteins (CaBP), such as oncomodulin and S100-P, which were found in the placenta [17,18,19,20,21]. Many studies support the role of PTH-rP in the placental Ca^2+^ transport; a fragment of PTH-rP is most likely responsible for stimulating Ca^2+^ transport via PTH-R1, a G-protein-coupled receptor [15]. Interestingly, PTH-rP is also secreted by smooth muscle cells in vascular beds, responding to a stretching by relaxing smooth muscle locally with a vasorelaxant effect [22,23]. Macgill et al., in 1997, demonstrated that PTH-rP and PTH infusion in pre-constricted placental cotyledons induced a significative vasodilatation of fetoplacental vessels in vitro [24].

PTH-rP was also found to be a cytotrophoblast apoptosis survival factor; in cell culture from term human placentas, PTH-rP evoked a rescue of both TNFα/IFNγ and staurosporine-induced apoptosis, with a significantly reduced rate of apoptosis [25]. Animal models demonstrated that PTH-rP is expressed in mouse embryonic and extraembryonic tissues from the late morula stage onwards, in the trophoblast cells in early pregnancy, and in the developing trophoectoderm cell lineage [26]. A PTH-rP gene knockout murine model demonstrated that null-PTH-rP gene pregnancies are associated with reduced trophoblast giant cells, reduced fetal weight, and reduced placental amino acid transport [27]. Wlodeck et al. in 2004 studied the effect of PTH-rP infusion in spontaneous hypertensive rat (SHR) models and they demonstrated that exogenous administration of PTH-rP did not have any effect on fetal growth, while PTH/PTH-rP receptor antagonism induced an increase in endogenous PTH-rP, which mediated increased fetal growth, possibly via autocrine actions [28].

PTH-R1 is a G-protein-coupled receptor that binds PTH and PTH-rP with similar affinity and can activate several intracellular signaling pathways, including those involving cAMP and Ca^2+^. The immediate consequences of activating PTH-R1 differ between cell types, and even between studies of the same cell lines [29]. Authors have demonstrated that the renal and bone PTH-R1 expression is upregulated in vitamin-D-deficient rats and by endotoxin, interleukin-2, dexamethasone, T3, and TGF. On the contrary, PTH, PTH-rP, angiotensin-II, IGF-1, PGE2, vitamin D, and chronic renal failure decrease its expression [30].

Recently, the relationship between bone metabolism and the glucose metabolism perturbations has been described [31]. Impairment of the calcium–vitamin D–PTH axis has been shown in patients with diabetes. In 1992, Ishida et al. reported that type 2 diabetics presented higher serum PTH-rP levels than control subjects [32]. Extensive studies have demonstrated that an increase in the cytosolic calcium is essential for glucose-stimulated insulin release and that PTH-rP induces insulin expression in pancreatic β-cells [8,33]. Furthermore, a recent study demonstrated the presence of insulin receptors INSR, IGF1R and IGF2R in the parathyroid tissue [34]. In 2007, Maghbooli et al. analyzed a cohort of 741 pregnancies and found that severe vitamin D deficiency (<25 nmol/L) was higher in women with a diagnosis of GDM compared to non-GDM women [35]. In contrast, recently Kramer et al. showed that vitamin D level was not associated with the insulin sensitivity or with the glucose tolerance status, but their data demonstrated a positive correlation between the prevalence of GDM with the increase of PTH levels [36].

To the best of our knowledge, this is the first study to provide a wide understanding of the placental PTH-rP and PTH-R1 expression in pregnancies complicated by GDM and to evaluate the relationship between this expression and the maternal, pregnancy, and neonatal outcomes. Our data show that placental PTH-rP and PTH-R1 expression are associated with peculiar pregnancy characteristics in the GDM patients. PTH-rP expression is associated with maternal elevated fasting glucose levels, maternal obesity, and Apgar score at 1 min < 7, while PTH-R1 is overexpressed in placentas from pregnancies with maternal elevated fasting glucose levels, lower ultrasound-estimated fetal weight, and lower fetoplacental ratio, suggesting the correlation between these markers, impaired glycemic control, and adverse pregnancy outcomes. Therefore, PTH-rP and PTH-R1 are more expressed in placentas from GDM patients with a phenotype characterized by increased insulin resistance due to metabolic syndrome, increased fasting glycemia, and normal glycemia after fuel load, rather than in placentas from GDM patients with impaired insulin secretion, which usually present normal fasting glycemia and abnormal increased glycemia after fuel load. These findings are coherent with already published data. Zhang et al. demonstrated that PTH-rP induces insulin expression by dephosphorylating c-Jun N-terminal kinases JNK1/2 in pancreatic beta-cells, while Roca-Rodríguez et al. found that PTH-rP mRNA expression in visceral adipose tissue seems to be related to obesity-associated insulin resistance [37,38]. In this perspective, we hypothesize that PTH-rP and PTH-R1 are upregulated in placentas from women with increased insulin resistance and metabolic syndrome, and their overexpression should be considered a compensative mechanism aiming to provide an increased calcium-mediated insulin sensitivity in women with GDM and increased insulin resistance. Interestingly, in extravillous trophoblast, PTH-rP and PTH-R1 correlated with different clinical parameters. It is unclear whether this difference may be due to common regulatory mechanisms in the placental expression of these two markers. Since PTH-rP expression decreases PTH-R1 expression, increased placental PTH-R1 may be associated with lower placental PTH-rP expression and, thus, lower fetal weight and fetoplacental ratio.

This is the first study to evaluate PTH-rP and PTH-R1 expression in term of placentas from pregnancies complicated by GDM and to correlate these data with maternal characteristics and pregnancy adverse outcomes. However, limited samples and the lack of a complete evaluation of PTH–vitamin D–calcium axis in these pregnancies (including PTH, PTH-rP, and PTH-r1 expression in maternal serum or umbilical cord blood) make it difficult to clearly understand the role of PTH-rP and PTH-R1 in the placental pathophysiology in pregnancies complicated by GDM.

## 5. Conclusions

Our data suggest that PTH-rP and PTH-R1 may be useful as markers of placental adaptation to maternal impaired glucose control and insulin resistance. More studies would be needed to confirm our hypothesis and to provide a comprehensive understanding of the interplay between calcium and glucose metabolism in pregnancies with gestational diabetes.

## Figures and Tables

**Table 1:** Maternal, obstetric, and neonatal characteristics of included women.

Pregnancy Characteristics	*n* = 78	*p* *
Maternal age (years)	35 (7)	0.233
Pregestational weight (kg)	68.51 (14.77)	<0.001
Pregestational BMI (kg/m^2^)	25.71 (5.91)	<0.001
Maternal BMI ≥ 30 kg/m^2^	15 (19.2%)	
Previous GDM	9 (11.1%)	
Nullipara	37 (47.4%)	
Insulin or metformin therapy	38 (48.8%)	
Third-trimester ultrasound-estimated fetal weight percentile	64.31 (28.24)	0.001
Gestational week at delivery (weeks)	38.93 (1.06)	0.030
Preterm birth	4 (5.1%)	
Vaginal delivery	52 (66.7%)	
Induction of labor	22 (28.2%)	
Urgent CS or OVD	9 (11.5%)	
Non-reassuring CTG	7 (7.1%)	
Neonatal sex (male)	38 (48.7%)	
Neonatal birthweight (g)	3240 (500)	0.256
Placental weight (g)	529 (151)	0.642
Fetoplacental weight ratio	6.17 (1.04)	0.017
Neonatal birthweight percentile	51.59 (30.56)	0.002
Neonatal LGA	11 (14.1%)	
Neonatal RDS	4 (5.1%)	
Apgar at 1 ≤ 7	4 (5.1%)	
Arterial cord blood pH ≤ 7.15	4 (5.1%)	
Neonatal admission to NICU or neonatal ward	5 (6.4%)	
Composite neonatal adverse outcome	11 (14.1%)	

Data are given as *n* (%), mean (standard deviation), or median (interquartile range). * Shapiro–Wilk test for normality distribution of continuous variables. BMI, body mass index; GDM, gestational diabetes; CS, caesarean section; OVD, operative vaginal delivery; CTG, cardiotocography; LGA, large for gestational age; RDS, respiratory distress syndrome; NICU, neonatal intensive care unit.

**Table 2:** PTH-rP and PTH-R1 immunochemistry positivity according to the placental layer.

Immunochemistry Expression	*n* = 78
PTH-rP extravillous cytotrophoblast	31 (39%)
PTH-rP syncytiotrophoblast	9 (11.5%)
PTH-rP villous cytotrophoblast	13 (16.7%)
PTH-rP decidua	29 (37.2%)
PTH-rP villous stroma	0 (0%)
PTH-R1 extravillous cytotrophoblast	52 (66.7%)
PTH-R1 syncytiotrophoblast	11 (14.1%)
PTH-R1 villous cytotrophoblast	41 (52.6%)
PTH-R1 decidua	58 (74.4%)
PTH-R1 villous stroma	0 (0%)

## Data Availability

The data that support the findings of this study are available from the corresponding author upon request.

## Supplementary Materials

The following are available online at https://www.mdpi.com/article/10.3390/diagnostics11081356/s1, Table S1: PTH-rP expression in extravillous cytotrophoblast according to maternal, pregnancy and neonatal characteristics, Table S2: PTH-R1 expression in extravillous cytotrophoblast according to maternal, pregnancy and neonatal characteristics, Table S3: PTH-rP expression in syncytiotrophoblast according to maternal, pregnancy and neonatal characteristics, Table S4: PTH-R1 expression in syncytiotrophoblast according to maternal, pregnancy and neonatal characteristics, Table S5: PTH-rP expression in villous cytotrophoblast according to maternal, pregnancy and neonatal characteristics, Table S6: PTH-rP expression in decidua according to maternal, pregnancy and neonatal characteristics, Table S7: PTH-R1 expression in villous cytotrophoblast according to maternal, pregnancy and neonatal characteristics, Table S8: PTH-R1 expression in decidua according to maternal, pregnancy and neonatal characteristics.
