# Supplementary material for: PTH-rP and PTH-R1 Expression in Placentas from Pregnancies Complicated by Gestational Diabetes: New Insights into the Pathophysiology of Hyperglycemia in Pregnancy
Source: Diagnostics (Basel). 2021 Jul 28;11(8):1356. doi: 10.3390/diagnostics11081356 (PMC8394866; doi:10.3390/diagnostics11081356)
Supplement: Supplementary file 1 [file diagnostics-11-01356-s001.zip › diagnostics-1288245-supplementary.pdf]

Table S1. PTHrp expression in extravillous cytotrophoblast according to maternal, pregnancy and neonatal characteristics

	PTH-rP extravillous cytotrophoblast positive (n=31)	PTH-rP extravillous cytotrophoblast negative (n=47)	p*
Maternal age (years)	35.52 (4.53)	34.96 (4.33)	0.586
Pregestational weight (kg)	67 (20)	65 (12)	0.591
Pregestational BMI (kg/m^2)	24.61 (7.51)	25.04 (7.71)	0.814
Maternal BMI $\geq$ 30kg/m^2	7 (21.4%)	9 (19.1%)	0.811
Previous GDM	2 (6.5%)	7 (14.9%)	0.304
Nullipara	15 (48.4%)	22 (46.8%)	0.891
Insulin or metformin therapy	14 (45.2%)	24 (52.2%)	0.546
OGTT 0	25 (80.6%)	25 (53.2%)	**0.013**
Third trimester ultrasound estimated fetal weight percentile	55 (56)	69 (45)	0.987
Gestational week at delivery (weeks)	39.00 (1.29)	39.14 (1.29)	0.114
Preterm birth	2 (6.5%)	2 (4.3%)	1.000
Vaginal delivery	20 (64.5%)	32 (68.1)	0.744
Induction of labor	10 (32.3%)	12(25.5%)	0.518
Urgent CS or OVD	3 (9.7%)	6 (12.8%)	1.000
Non-reassuring CTG	2 (6.5%)	3 (6.4%)	1.000
Neonatal sex (male)	15 (48.4%)	23 (48.9%)	0.962
Neonatal birthweight (g)	3213.23 (286.19)	3226.70 (479.0.)	0.888
Placental weight (g)	525.01 (105.35)	546.07 (127.92)	0.449
Fetoplacental weight ratio	5.95 (1.44)	5.89 (1.73)	0.411
Neonatal birthweight percentile	50 (38)	49 (64)	0.557
Neonatal LGA	4 (12.%)	7 (14.9%)	1.000
Neonatal RDS	0 (0%)	4 8.5%)	0.147
Apgar at 1 <7	1 (3.2%)	3 (6.4%)	1.000
Arterial cord blood pH <7.15			
Neonatal admission to NICU or neonatal ward	2 (6.5%)	3 (6.5%)	0.990
Composite neonatal adverse outcome	5 (16.1%)	6 (12.8%)	0.676

Dichotomous variables are given as n (%); continuous parametrical data are givens as mean (standard deviation); continuous non-parametrical data are given as median (interquartile range). *Student t-test for continuous parametrical data; Kruskal-Wallis test for continuous non-parametrical data ; Pearson Chi-square test for dichotomous variables.

Table S2. PTH-R1 expression in extravillous cytotrophoblast according to maternal, pregnancy and neonatal characteristics

	PTH-R1 extravillous cytotrophoblast positive (n=52)	PTH-R1 extravillous cytotrophoblast negative (n=26)	p
Maternal age (years)	34.87 (4.21)	35.81 (4.77)	0.375
Pregestational weight (kg)	66.50 (21)	65.50 (11)	0.280
Pregestational BMI (kg/m^2)	25.34 (7.94)	24.38 (5.70)	0.409
Maternal BMI $\geq$ 30kg/m^2	11 (22%)	4 (16%)	0.761
Previous GDM	7 (13.5%)	2 (7.7%)	0.710
Nullipara	30 (57.7%)	11 (42.3%)	0.200
Insulin or metformin therapy	23 (45.1%)	15 (57.7%)	0.296
OGTT 0	37 (71.2%)	13 (50%)	**0.066**
Third trimester ultrasound estimated fetal weight percentile	55 (48.75)	79.5 (28)	**0.022**
Gestational week at delivery (weeks)	39.14 (1.39)	39.14 (0.61)	0.746
Preterm birth	2 (3.8%)	2 (7.7%)	0.597
Vaginal delivery	34 (65.4%)	18 (69.2%)	0.734
Induction of labor	18 (34.6%)	4 (15.4%)	0.109
Urgent CS or OVD	7 (13.5%)	2 (7.7%)	0.710
Non-reassuring CTG	5 (9.6%)	0	**0.163**
Neonatal sex (male)	27 (51.9%)	11 (42.3%)	0.423
Neonatal birthweight (g)	3211.15 (389.79)	3241.73 (458.29)	0.759
Placental weight (g)	547.54 (121.38)	518.01 (114.44)	0.305
Fetoplacental weight ratio	5.71 (1.53)	6.34 (1.20)	**0.042**
Neonatal birthweight percentile	47 (51.75)	62 (65.25)	0.754
Neonatal LGA	6 (11.5%)	5 (19.2%)	0.357
Neonatal RDS	2 (3.8%)	2 (7.7%)	0.597
Apgar at 1 <7	2 (3.8%)	2 (7.7%)	0.597
Arterial cord blood pH <7.15			
Neonatal admission to NICU or neonatal ward	3 (5.9%)	2 (7.7%)	1.000
Composite neonatal adverse outcome	8 (15.4%)	3 (11.5%)	0.743

Dichotomous variables are given as n (%); continuous parametrical data are givens as mean (standard deviation); continuous non-parametrical data are given as median (interquartile range). *Student t-test for continuous parametrical data; Kruskal-Wallis test for continuous non-parametrical data ; Pearson Chi-square test for dichotomous variables.

Table S3. PTHrp expression in syncytiotrophoblast according to maternal, pregnancy and neonatal characteristics

	PTH-rP syncytiotrophoblast positive (n=9)	PTH-rP syncytiotrophoblast negative (n=69)	p
Maternal age (years)	36.11 (4.37)	35.06 (4.41)	0.502
Pregestational weight (kg)	70 (35)	65 (14)	0.551
Pregestational BMI (kg/m^2)	24.54 (13.06)	25 (7.04)	0.608
Maternal BMI $\geq$ 30kg/m^2	4 (44.4%)	11 (16.7%)	**0.072**
Previous GDM	2 (22.2%)	7 (10.1%)	0.277
Nullipara	2 (22.2%)	35 (50.7%)	0.159
Insulin or metformin therapy	5 (55.6%)	33 (48.5%)	0.737
OGTT 0	7 (77.8)	43 (62.3%)	0.477
Third trimester ultrasound estimated fetal weight percentile	52 (63)	68 (46.50)	0.423
Gestational week at delivery (weeks)	39 (1.36)	39.14 (1.29)	0.875
Preterm birth	0 (0%)	4 (5.8%)	1.000
Vaginal delivery	4 (44.4%)	48 (69.8%)	0.151
Induction of labor	3 (33.3%)	19 (27.5%)	0.706
Urgent CS or OVD	1 (11.1%)	8 (11.6%)	1.000
Non-reassuring CTG	1 (11.1%)	4 (5.8%)	0.468
Neonatal sex (male)	5 (55.6%)	33 (47.8%)	0.734
Neonatal birthweight (g)	3090 (350.59)	3238.48 (417.57)	0.311
Placental weight (g)	514.86 (123.51)	540.68 (119.22)	0.544
Fetoplacental weight ratio	5.74 (1.85)	5.94 (1.52)	0.808
Neonatal birthweight percentile	29 (47.5)	51 (55)	0.123
Neonatal LGA	0 (0%)	11 (15.9%)	0.344
Neonatal RDS	1 (11.1%)	3 (4.3%)	0.394
Apgar at 1 $\leq$7	2 (22.2%)	2 (2.9%)	**0.063**
Arterial cord blood pH <7.15			
Neonatal admission to NICU or neonatal ward	1 (11.1%)	4 (5.9%)	0.472
Composite neonatal adverse outcome	2 (22.2%)	9 (13.0%)	0.607

Dichotomous variables are given as n (%); continuous parametrical data are givens as mean (standard deviation); continuous non-parametrical data are given as median (interquartile range). *Student t-test for continuous parametrical data; Kruskal-Wallis test for continuous non-parametrical data ; Pearson Chi-square test for dichotomous variables.

Table S4. PTH-R1 expression in syncytiotrophoblast according to maternal, pregnancy and neonatal characteristics

	PTH-R1 syncytiotrophoblast (n=11)	PTH-R1 syncytiotrophoblast (n=67)	p
Maternal age (years)	35.82 (3.40)	35.07 (4.54)	0.606
Pregestational weight (kg)	68 (29)	66 (15)	0.785
Pregestational BMI (kg/m^2)	25.98 (9.17)	24.61 (7.12)	0.858
Maternal BMI $\geq$ 30kg/m^2	2 (20%)	13 (20%)	1.000
Previous GDM	3 (27.3%)	6 (9%)	0.110
Nullipara	9 (81.8%)	32 (47.8%)	0.138
Insulin or metformin therapy	6 (54.5%)	34 (51.5%)	0.710
OGTT 0	9 (81.8%)	41 (61.2%)	0.310
Third trimester ultrasound estimated fetal weight percentile	70 (54.75)	68 (48)	0.721
Gestational week at delivery (weeks)	38.64 (1.71)	39.14 (1.07)	0.392
Preterm birth	0 (0%)	4 (6%)	1.000
Vaginal delivery	5 (45.5%)	47 (70.1%)	0.107
Induction of labor	3 (27.3%)	19 (28.4%)	1.000
Urgent CS or OVD	1 (9.1%)	8 (11.9%)	1.000
Non-reassuring CTG	1 (9.1%)	6	0.543
Neonatal sex (male)	3 (27.3%)	35 (52.2%)	0.194
Neonatal birthweight (g)	3264.54 (356.89)	3214.25 (421.37)	0.710
Placental weight (g)	579.25 (113.88)	530.88 (119.48)	0.214
Fetoplacental weight ratio	5.83 (1.19)	5.95 (1.54)	0.318
Neonatal birthweight percentile	63.50 (43.75)	44.50 (53.75)	0.157
Neonatal LGA	3 (27.3%)	8 (11.9%)	0.182
Neonatal RDS	1 (9.1%)	3 (4.5%)	0.463
Apgar at 1 <7	1 (9.1%)	3 (4.5%)	0.463
Arterial cord blood pH <7.15			
Neonatal admission to NICU or neonatal ward	1 (9.1%)	4 (6.1%)	0.548
Composite neonatal adverse outcome	1 (9.1%)	10 (14.9%)	1.000

Dichotomous variables are given as n (%); continuous parametrical data are givens as mean (standard deviation); continuous non-parametrical data are given as median (interquartile range). *Student t-test for continuous parametrical data; Kruskal-Wallis test for continuous non-parametrical data ; Pearson Chi-square test for dichotomous variables.

Table S5. PTHrp expression in villous cytotrophoblast according to maternal, pregnancy and neonatal characteristics

	PTH-rP villous cytotrophoblast positive (n=13)	PTH-rP villous cytotrophoblast negative (n=65)	p
Maternal age (years)	36.69 (4.13)	34.88 (4.41)	0.175
Pregestational weight (kg)	65 (25)	67 (14)	0.520
Pregestational BMI (kg/m^2)	24.54 (10.99)	25.04 (6.96)	0.301
Maternal BMI $\geq$ 30kg/m^2	3 (25%)	12 (19%)	0.697
Previous GDM	1 (7.7%)	8 (12.3%)	1.000
Nullipara	7 (53.8%)	34 (52.3%)	0.919
Insulin or metformin therapy	5 (38.5%)	33 (51.6%)	0.389
OGTT 0	10 (76.9%)	40 (61.5%)	0.357
Third trimester ultrasound estimated fetal weight percentile	55 (43)	69 (53)	0.938
Gestational week at delivery (weeks)	39.14 (1.00)	39.14 (1.29)	0.824
Preterm birth	1 (7.7%)	3 (4.6%)	0.525
Vaginal delivery	8 (61.5%)	44 (67.7%)	0.667
Induction of labor	4 (30.8%)	18 (27.7%)	1.000
Urgent CS or OVD	1 (7.7%)	8 (12.3%)	1.000
Non-reassuring CTG	1 (7.7%)	4 (6.2%)	1.000
Neonatal sex (male)	4 (30.8%)	34 (52.3%)	0.226
Neonatal birthweight (g)	3207.30 (204.49)	3224.15 (442.14)	0.894
Placental weight (g)	532.75 (101.69)	538.69 (123.08)	0.871
Fetoplacental weight ratio	6.07 (1.90)	5.92 (1.52)	0.634
Neonatal birthweight percentile	44 (22)	52 (59)	0.841
Neonatal LGA	1 (7.7%)	10 (15.4%)	0.679
Neonatal RDS	0 (0%)	4 (6.2%)	1.000
Apgar at 1 <7	0 (0%)	4 (6.2%)	1.000
Arterial cord blood pH <7.15			
Neonatal admission to NICU or neonatal ward	0 (0%)	5 (7.8%)	0.582
Composite neonatal adverse outcome	1 (7.7%)	10 (15.4%)	0.679

Dichotomous variables are given as n (%); continuous parametrical data are givens as mean (standard deviation); continuous non-parametrical data are given as median (interquartile range). *Student t-test for continuous parametrical data; Kruskal-Wallis test for continuous non-parametrical data ; Pearson Chi-square test for dichotomous variables.

Table S6. PTHrP expression in decidua according to maternal, pregnancy and neonatal characteristics

	PTH-rP Decidua positive (n=29)	PTH-rP Decidua negative (n=49)	p
Maternal age (years)	35.48 (5.53)	35.01 (3.61)	0.642
Pregestational weight (kg)	66 (16)	66 (18)	0.938
Pregestational BMI (kg/m^2)	24.60 (6.44)	25 (7.89)	
Maternal BMI $\geq$ 30kg/m^2	6 (22.2%)	9 (18.8%)	0.718
Previous GDM	3 (10.3%)	6 (12.2%)	1.000
Nullipara	17 (58.6%)	24 (49.0%)	0.410
Insulin or metformin therapy	13 (44.8%)	25 (52.1%)	0.537
OGTT 0	17 (58.6%)	33 (67.3%)	0.437
Third trimester ultrasound estimated fetal weight percentile	75 (57)	63 (44)	0.138
Gestational week at delivery (weeks)	39 (1.29)	39.14 (1.29)	0.261
Preterm birth	2 (6.9%)	2 (4.1%)	0.625
Vaginal delivery	19 (65.5%)	33 (67.3%)	0.868
Induction of labor	9 (31%)	13 (26.5%)	0.669
Urgent CS or OVD	2 (6.9%)	7 (14.3%)	0.471
Non-reassuring CTG	1 (3.4%)	4 (8.2%)	0.646
Neonatal sex (male)	14 (48.3%)	24 (49%)	0.952
Neonatal birthweight (g)	3264.65 (321.61)	3195.71 (457.20)	0.478
Placental weight (g)	546.05 (102.84)	532.76 (128.66)	0.637
Fetoplacental weight ratio	5.85 (1.56)	6.21 (1.52)	0.668
Neonatal birthweight percentile	58 (45)	44 (58)	0.273
Neonatal LGA	4 (13.8%)	7 (14.3%)	1.000
Neonatal RDS	1 (3.4%)	3 (6.1%)	1.000
Apgar at 1 <7	2 (6.9%)	2 (4.1%)	0.625
Arterial cord blood pH <7.15			
Neonatal admission to NICU or neonatal ward	2 (6.9%)	3 (6.2%)	1.000
Composite neonatal adverse outcome	4 (13.8%)	7 (14.3%)	1.000

Dichotomous variables are given as n (%); continuous parametrical data are givens as mean (standard deviation); continuous non-parametrical data are given as median (interquartile range).

*Student t-test for continuous parametrical data; Kruskal-Wallis test for continuous non-parametrical data ; Pearson Chi-square test for dichotomous variables.

Table S7. PTH-R1 expression in villous cytotrophoblast according to maternal, pregnancy and neonatal characteristics

	PTH-R1 villous cytotrophoblast (n=41)	PTH-R1 villous cytotrophoblast (n=37)	p
Maternal age (years)	34.98 (4.55)	35.41 (4.26)	0.669
Pregestational weight (kg)	67 (22)	65.50 (15)	0.983
Pregestational BMI (kg/m^2)	25.17 (6.31)	24.38 (7.92)	0.703
Maternal BMI $\geq$ 30kg/m^2	8 (20.5%)	7 (19.4%)	0.908
Previous GDM	6 (14.6%)	3 (8.1%)	0.487
Nullipara	23 (56.1%)	18 (48.6%)	0.511
Insulin or metformin therapy	22 (53.7%)	16 (44.4%)	0.420
OGTT 0	26 (63.4%)	24 (64.9%)	0.894
Third trimester ultrasound estimated fetal weight percentile	69.50 (52.25)	63 (47.75)	0.289
Gestational week at delivery (weeks)	39.14 (1.32)	39.14 (1.43)	0.602
Preterm birth	1 (2.4%)	3 (8.1%)	0.341
Vaginal delivery	28 (68.3%)	24 (64.9%)	0.748
Induction of labor	13 (31.7%)	9 (24.3%)	0.469
Urgent CS or OVD	5 (12.2%)	4 (10.8%)	1.000
Non-reassuring CTG	3 (7.3%)	2 (5.4%)	1.000
Neonatal sex (male)	17 (41.5%)	21 (56.8%)	0.177
Neonatal birthweight (g)	3212.92 (374.57)	3230.67 (453.32)	0.850
Placental weight (g)	533.41 (130.04)	542.45 (107.49)	0.740
Fetoplacental weight ratio	6.07 (2.04)	5.91 (1.23)	0.545
Neonatal birthweight percentile	50 (56.75)	46 (54.25)	0.904
Neonatal LGA	6 (14.6%)	5 (13.5%)	0.887
Neonatal RDS	3 (7.3%)	1 (2.7%)	0.617
Apgar at 1 <7	3 (7.3%)	1 (2.7%)	0.617
Arterial cord blood pH <7.15			
Neonatal admission to NICU or neonatal ward	4 (10%)	1 (2.7%)	0.360
Composite neonatal adverse outcome	5 (12.2%)	6 (16.2%)	0.610

Dichotomous variables are given as n (%); continuous parametrical data are givens as mean (standard deviation); continuous non-parametrical data are given as median (interquartile range). *Student t-test for continuous parametrical data; Kruskal-Wallis test for continuous non-parametrical data ; Pearson Chi-square test for dichotomous variables.

Table S8. PTH-R1 expression in decidua according to maternal, pregnancy and neonatal characteristics

	PTH-R1 Decidua positive (n=73)	PTH-R1 Decidua negative (n=26)	p
Maternal age (years)	35.10 (4.49)	35.40 (4.17)	0.796
Pregestational weight (kg)	67 (20)	64.50 (15)	0.611
Pregestational BMI (kg/m^2)	25.17 (7.94)	24.24 (6.42)	0.491
Maternal BMI $\geq$ 30kg/m^2	13 (22.8%)	2 (11.1%)	0.499
Previous GDM	7 (12.1%)	2 (10%)	1.000
Nullipara	33 (56.9%)	8 (40%)	0.192
Insulin or metformin therapy	26 (44.8%)	12 (63.2%)	0.165
OGTT 0	36 (62.1%)	14 (70%)	0.524
Third trimester ultrasound estimated fetal weight percentile	68.50 (55.25)	67 (30.25)	0.839
Gestational week at delivery (weeks)	39.14 (1.50)	39.14 (1.29)	0.256
Preterm birth	2 (3.4%)	2 (10%)	0.270
Vaginal delivery	40 (69%)	12 (60%)	0.463
Induction of labor	17 (29.3%)	5 (25%	0.712
Urgent CS or OVD	6 (10.3%)	3 (15%)	0.687
Non-reassuring CTG	3 (5.2%)	2 (10%)	0.598
Neonatal sex (male)	28 (48.3%)	10 (50%)	0.894
Neonatal birthweight (g)	3228.10 (376.39)	3201.75 (509.41)	0.807
Placental weight (g)	530.56 (116.84)	558.42 (126.54)	0.371
Fetoplacental weight ratio	6.22 (1.44)	5.90 (1.27)	0.170
Neonatal birthweight percentile	51.50 (56.25)	38.50 (54)	0.689
Neonatal LGA	7 (12.1%)	4 (20%)	0.459
Neonatal RDS	3 (5.2%)	1 (5%)	1.000
Apgar at 1 <7	4 (7%)	0 (0%)	0.567
Arterial cord blood pH <7.15			
Neonatal admission to NICU or neonatal ward	4 (7%)	1 (5%)	1.000
Composite neonatal adverse outcome	8 (13.8%)	3 (15%)	1.000

Dichotomous variables are given as n (%); continuous parametrical data are givens as mean (standard deviation); continuous non-parametrical data are given as median (interquartile range).

*Student t-test for continuous parametrical data; Kruskal-Wallis test for continuous non-parametrical data ; Pearson Chi-square test for dichotomous variables.
